# Related Melioidosis Cases with Unknown Exposure Source, Georgia, USA, 1983–2024

**DOI:** 10.3201/eid3109.250804

**Published:** 2025-09

**Authors:** Skyler Brennan, Julie M. Thompson, Christopher A. Gulvik, Taylor K. Paisie, Mindy Glass Elrod, Jay E. Gee, Caroline A. Schrodt, Katherine M. DeBord, Brian T. Richardson, Cherie Drenzek, William A. Bower, Alex R. Hoffmaster, Zachary P. Weiner, Caitlin M. Cossaboom, Julie Gabel

**Affiliations:** Georgia Department of Public Health, Atlanta, Georgia, USA (S. Brennan, C. Drenzek, J. Gabel); Centers for Disease Control and Prevention, Atlanta (J.M. Thompson, C.A. Gulvik, T.K. Paisie, M.G. Elrod, J.E. Gee, C.A. Schrodt, K.M. DeBord, B.T. Richardson, Jr., W.A. Bower, A.R. Hoffmaster, Z.P. Weiner, C.M. Cossaboom)

**Keywords:** Melioidosis, *Burkholderia pseudomallei*, bacteria, sepsis, Georgia, United States, whole-genome sequencing

## Abstract

We identified 4 cases of presumptive autochthonous melioidosis during 1983–2024 in Georgia, USA. Epidemiologic investigation identified no recent international travel before illness; all cases were geographically linked, and 3 patients became ill after a severe weather event. Bioinformatic analyses revealed *Burkholderia pseudomallei* genome sequences were highly related, suggesting a shared exposure.

Melioidosis is a potentially severe disease caused by the gram-negative environmental bacterium *Burkholderia pseudomallei* (*1*), which is predominantly found in tropical and subtropical regions. The median incubation is 4 days (total range 1–21 days) after exposure. Clinical manifestations vary, and infection can cause local or disseminated disease, including fulminant sepsis. Mortality ranges from <10% with early recognition and access to intensive care to >40% without treatment (*1*,*2*). Melioidosis does not develop from *B. pseudomallei* exposure in most persons, but comorbidities such as diabetes, which is prevalent in the southeastern United States (*3*), increase the risk (*2*).

Most melioidosis cases in the United States are associated with travel to endemic areas, but some were domestically acquired after exposure to imported household products (*4*,*5*). In 2022, three human cases were genetically linked to environmental *B. pseudomallei* isolated from the US Gulf Coast (*6*). Predictive modeling studies suggest environmental conditions in the southeastern United States are suitable for *B. pseudomallei* (*7*). In endemic regions, infections increase during the rainy season and after severe weather events such as hurricanes (*1*,*2*,*8*,*9*). The Atlantic hurricane season spans from June 1–November 30 annually (*10*).

## The Study

On September 26, 2024, category 4 Hurricane Helene made landfall in Georgia, USA (Table), resulting in heavy rainfall, flooding, and windspeeds reaching 100 mph. On October 9, blood cultures from 2 patients in Georgia were presumptive positive for *B. pseudomallei*. The Georgia Department of Public Health discovered the patients shared a common worksite with exposure to mud, dust, wind, and 10 inches of rain.

**Table:** Characteristics, clinical findings, and timeline of events among 4 patients with related melioidosis infections, Georgia, USA, 1983–2024*

Characteristics	Patient 1	Patient 2	Patient 3	Patient 4
Preceding weather event (strength†)	Hurricane Helene (Category 4)		Hurricane Hugo (Category 4)	None
Weather event date	2024 Sep 26	2024 Sep 26	1989 Sep 22	None
Symptom onset date(s)	2024 Sep 28	2024 Sep 29	1989 Oct 5	Unknown
Hospitalization admission date	2024 Oct 2	2024 Oct 1	Unknown	Unknown
Laboratory findings at admission (reference range)				
Leukocyte count (4–11 × 10^9^ cells/L)	14.98	5.92	Unknown	Unknown
Neutrophils (39.4%–72.5%)	77.8%	82.5%	Unknown	Unknown
Platelet count (140–400 × 10^9^/L)	162	73	Unknown	Unknown
AST (15–37 U/L)	37	193	Unknown	Unknown
ALT (16–61 U/L)	76	79	Unknown	Unknown
Imaging findings at admission	Chest radiograph: unremarkable; persistent nodule/density in left mid-lung zone. CT abdomen and pelvis with IV contrast: unremarkable	Chest radiograph: pneumonia with nodule in left lung, suspected bronchogenic neoplasm. CT abdomen and pelvis without contrast: unremarkable	Unknown	Unknown
Comorbidities	None identified	None identified	Diabetes	None identified
Outcome	Discharged	Discharged	Died	Died
Discharge or death date	2024 Oct 16	2024 Oct 20	1989 Oct	1983 Oct
Hospital readmission date	None	2024 Nov 9	None	None
Laboratory findings at readmission (reference range)				
Leukocyte count (4–11 × 10^9^ cells/L)	NA	12.5	NA	NA
AST (15–37 U/L)	NA	705	NA	NA
ALT (16–61 U/L)	NA	500	NA	NA
Imaging findings at readmission	NA	Chest radiograph: negative for consolidation. CT abdomen and pelvis without contrast: enlarged, edematous prostate, consistent with prostatitis	NA	NA
Readmission outcome	NA	Discharged	NA	NA
Readmission discharge or death date	NA	2024 Nov 15	NA	NA
International travel history (year)	NA	Bahamas (2022)	Unknown	South Korea (1950s), Vietnam (1960s)
United States military service history	NA	None	World War II	World War II, Korean War, Vietnam War

*ALT, alanine aminotransferase; AST, aspartate transferase; CT, computed tomography; IV, intravenous; NA, not applicable. †Hurricane category according to the Saffir-Simpson Hurricane Wind Scale (*10*).

Patient 1, a man in his 50s with no comorbidities, no reported international travel, and no US military service, reported working in muddy conditions on September 26, 2024. He performed routine vehicle inspections involving checking air pressure of vehicle tires, high-pressure hosing, and physical contact with mud. He became ill on September 28 and was hospitalized October 2 with fever, chills, weakness, and shortness of breath. Chest, abdominal, and pelvic imaging were unremarkable. Healthcare providers diagnosed severe sepsis. After the presumptive positive blood culture, treatment with 30 days of intravenous meropenem and oral doxycycline began on October 9, and then 3 months of oral doxycycline (*11*) was initiated. The patient was discharged on October 16. 

Patient 2, a man in his 60s with no comorbidities, travel to the Bahamas in 2022, and no US military service, reported operating heavy equipment at the worksite during September 26–29, 2024, and frequent contact with soil. We could not confirm if he performed high-pressure hosing. He became ill on September 29 and was hospitalized October 1 for fever, chills, weakness, confusion, and body aches. Chest imaging revealed pneumonia. Results of abdominal and pelvic imaging were unremarkable. Healthcare providers diagnosed severe sepsis without septic shock. After presumptive positive blood cultures, treatment with 30 days of intravenous ceftazidime and oral doxycycline began on October 9, and then 3 months of oral doxycycline (*11*) was initiated. The patient was discharged October 10. 

Patient 2 was readmitted on November 9 for fatigue, malaise, weakness, and shortness of breath. Abdominal and pelvic imaging suggested prostatitis. The patient received 6 weeks of intravenous meropenem and oral doxycycline beginning November 15 and then 3 months of oral doxycycline. Blood and urine cultures were positive for *B. pseudomallei*. Repeat blood cultures had no growth by November 12. The patient was discharged on November 15 and was lost to follow-up.

**Figure 1:**
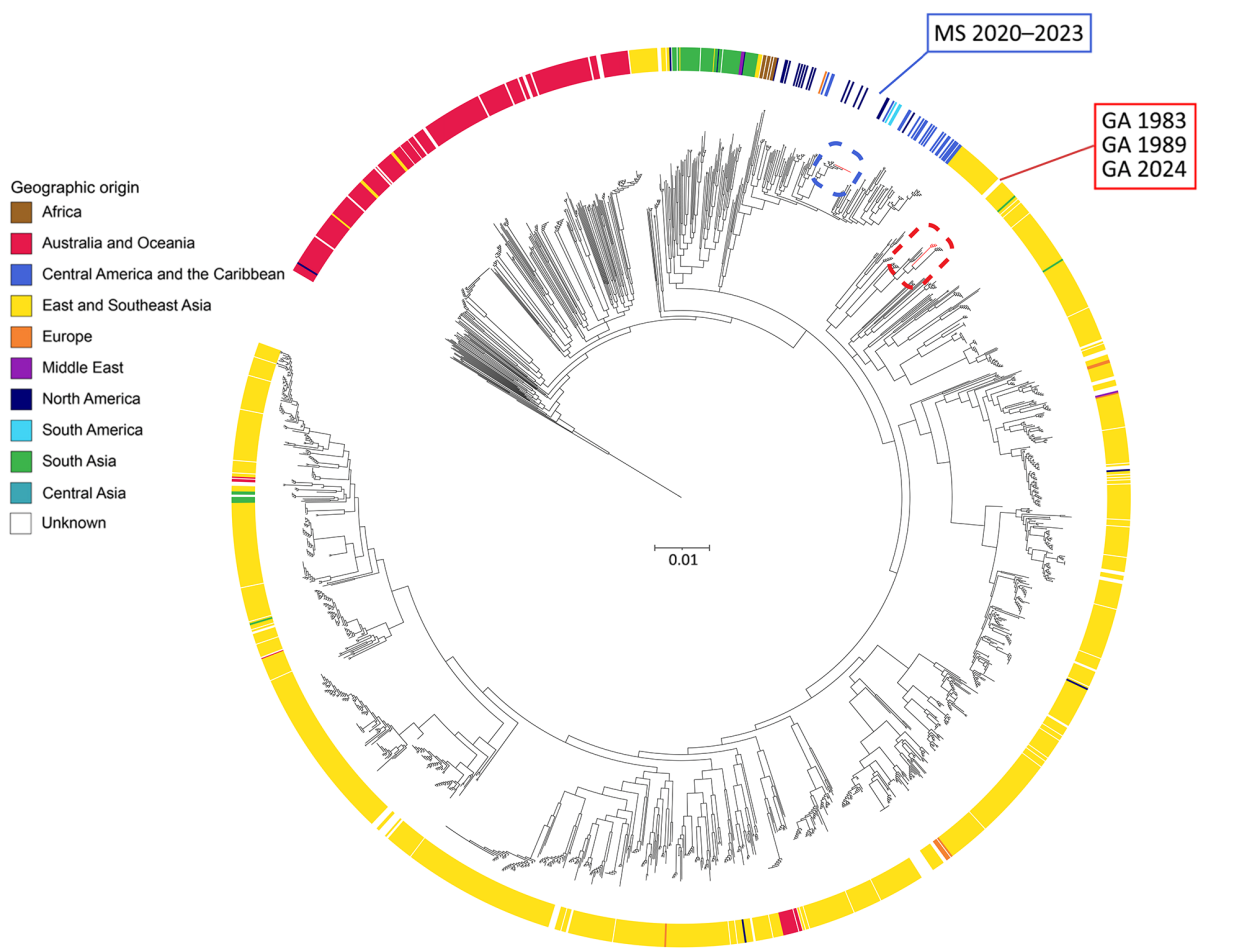
Global maximum-likelihood phylogenetic tree of core-genome single-nucleotide polymorphisms comparing new isolate genomes from 4 related melioidosis cases with unknown exposure source, Georgia, USA, 1983–2024 (red dashed red rectangle), with all *B. pseudomallei* genomes from the Center for Disease Control and Prevention’s internal and National Center for Biotechnology Information’s worldwide RefSeq databases as of August 6, 2024 (n = 1,976 genomes). Strain MSHR668 was used as an outgroup. New isolate genomes are distinct from *B. pseudomallei* genomes isolated from the environment in Mississippi (dashed blue rectangle). Isolates with a reported geographic origin are associated with their country of origin and rings are colored according to definitions listed in The World Factbook (https://www.cia.gov/the-world-factbook). Scale bar units represent substitutions per site from a 22% core alignment against *B. pseudomallei* strain 110 (7.78 Mbp size, RefSeq accession no. GCF_001905265.1).

We confirmed the patient isolates as *B. pseudomallei* by using the Centers for Disease Control and Prevention’s (CDC) Laboratory Response Network algorithm. We extracted isolate DNA for whole-genome sequencing (WGS) as previously described (*6*). Multilocus sequence typing (ST) indicated all isolates were ST41, which is associated with strains of Eastern Hemisphere origin. Phylogenetic analysis indicated the new patient-derived genomes grouped with strains from East and Southeast Asia (Figure 1) (*6*).

**Figure 2:**
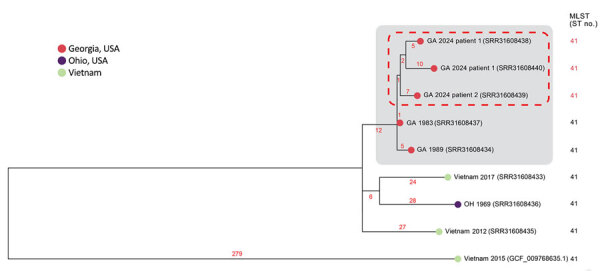
Subpanel maximum-likelihood phylogeny of refined mutation-only core single-nucleotide polymorphism sites of isolate genomes from 4 related melioidosis cases with unknown exposure source, Georgia, USA, 1983–2024, which includes the clinical isolates from the 2 patients from Georgia in 2024 (red dashed rectangle). *B. pseudomallei* genome sequences from the patient isolates were highly related to each other and to 2 other patients from Georgia in 1983 and 1989 (gray rectangle). Those genomes clustered most closely with genomes from Southeast Asia, particularly from Vietnam. Tree leaves contain year of isolation and National Center for Biotechnology Information sequence read archive and RefSeq accession numbers that correspond to each isolate. Branch numbers indicate the number of single nucleotide polymorphisms per site. MLST, multilocus sequence type; ST, sequence type.

We conducted WGS on 7 *B. pseudomallei* isolates from CDC’s multidecade surveillance archive on the basis of ST or geographic proximity to the 2024 cases for higher resolution analysis (*6*). Five isolates were ST41 and clustered with the 2024 patient-derived genomes (*12*,*13*). Two isolates were from 1 person who traveled to Vietnam, 2 were from 2 retired US military members, and data were limited for 1 (Figure 2). The bacterial genomes from the 2024 patients were highly related to each other and to the 2 from the retired US military members (<20 single-nucleotide polymorphisms apart across all genomes). One publicly available ST41 genome related to this cluster was from a patient who traveled to Vietnam.

We requested medical and military service records from the National Archives and Records Administration for the 2 retired service members, who died from melioidosis in 1989 and 1983. At the time of their deaths, both lived in the same county in Georgia as the 2024 patients. Records were incomplete for patient 3, who died in October 1989. He was a US Army and Air Force Veteran who served in World War II and had no records of service in Vietnam. He was hospitalized at a Veterans Affairs facility in Georgia before his death. Patient 4, who died in October 1983, was a US Navy and Army veteran who served in World War II, the Korean War, and the Vietnam War. For 20 years, he worked on a military base located <1 mile from the shared worksite of the 2024 patients. Records indicate patient 3 retired 36 years before his death and patient 4 retired 12 years before his death. Weather records indicate no hurricanes affected the area in 1983. In September 1989, Hurricane Hugo made landfall in the United States as a Category 4 storm and affected this area of Georgia with 3–5 inches of rain.

## Conclusions

This investigation identified 4 presumptive autochthonous human melioidosis cases in Georgia, USA. Without leveraging historical surveillance isolates archived at CDC, we would have concluded the 2024 cases represented a potential new local or imported exposure. However, the relatedness of patient-derived isolates and the close geographic proximity of all 4 patients in Georgia are strongly suggestive of a shared, locally acquired environmental exposure, dating back to the 1980s. Only patient 4 was known to have traveled to Southeast Asia in his lifetime. Although activation of *B. pseudomallei* from latency 2 decades after exposure during the Vietnam War is plausible, it is rare (*14*). Further, retrospective review of available medical records from patients 3 and 4 revealed no earlier illnesses or hospitalizations consistent with melioidosis, and reactivation would not explain the epidemiologic and WGS links to the other cases. Persistence of a single ST of *B. pseudomallei* in the environment over several decades has been reported (*15*). Introduction and persistence of *B. pseudomallei* in the environment from repatriation of personnel or equipment associated with the Vietnam War is possible, but other sources of environmental introduction or local exposure cannot be ruled out. Isolation of *B. pseudomallei* from the environment is needed to determine local endemicity and characterize the public health risk.

Trimethoprim/sulfamethoxazole (TMP/SMX) is the recommended first-line antimicrobial drug for *B. pseudomallei* infection; doxycycline is indicated with TMP/SMX intolerance. Relapses are more common with doxycycline compared with TMP/SMX (*11*). 

All 4 patients from Georgia became ill or died in September and October. High wind speeds, rain, or flooding associated with hurricanes might have contributed to infections in 3 of the patients. Because hurricanes regularly affect the US (*10*), increased knowledge of melioidosis among healthcare providers is needed, particularly if patients have contact with floodwater, mud, or debris. Persons should wear waterproof clothing or boots, cover wounds if they cannot avoid floodwater, and follow local safety guidance during hurricanes.
